# Chemical composition, antimicrobial, and lipase enzyme activity of essential oil and solvent extracts from Serapias orientalis subsp. orientalis

**DOI:** 10.3906/kim-2005-51

**Published:** 2020-12-16

**Authors:** İshak ERİK, Gözde KILIÇ, Elif ÖZTÜRK, Şengül Alpay KARAOĞLU, Nurettin YAYLI

**Affiliations:** 1 Department of Pharmacognosy, Faculty of Pharmacy, Karadeniz Technical University, Trabzon Turkey; 2 Institute of Health Sciences, Karadeniz Technical University, Trabzon Turkey; 3 Departments of Nutrition and Dietetics, Faculty of Health Science, Karadeniz Technical University, Trabzon Turkey; 4 Department of Biology, Faculty of Science, Recep Tayyip Erdoğan University, Rize Turkey

**Keywords:** *Serapias orientalis* subsp. *orientalis*, volatile compounds, antimicrobial, lipase inhibition

## Abstract

The volatile components of essential oil (EO), SPME, and SPME of solvent extracts ( *n* -hexane, methanol, and water) obtained from fresh *Serapias orientalis* subsp. *orientalis* ( *Soo* ) were analyzed by GC-FID/MS. EO of *Soo* gave 11 compounds in the percentage of 99.97%; capronaldehyde (37.01%), 2-( *E* )-hexenal (23.19%), and *n* -nonanal (19.05%) were found to be major constituents. SPME GC-FID/MS analyses of fresh plant and solvent extracts of *Soo* revealed 7, 12, 7, and 4 compounds within the range of 99.7% to 99.9%. Limonene (76.5%, 41.7%, and 61.3%) was the major compound in SPMEs of the *n* -hexane and methanol extracts. *α* -Methoxy- *p* -cresol (52.9%) was the main component in its water extract. The antimicrobial activity of EO and the solvent extracts of *Soo* were screened against 9microorganisms. EO showed the best activity against *Mycobacterium smegmatis* , with 79.5 µg/mL MIC value. The *n* -hexane, methanol, and water extracts were the most active against the *Staphylococcus aureus* within the range of 81.25–125.0 µg/mL (MIC). IC _50_ values for the lipase enzyme inhibitory activity of EO and solvent extracts ( *n* -hexane, methanol, and water) were determined to be 59.87 µg/mL, 64.03 µg/mL, 101.91 µg/mL, and 121.24 µg/mL, respectively.

## 1. Introduction

Orchidaceae is among the richest plant families, with more than 25,000 species worldwide. *Serapias* L. is a genus of the Orchidaceae family which occurs from the eastern–central and eastern Mediterranean to the western Transcaucasusand is represented by 30 common taxa [1–3]. There are 23 species of Orchidaceae and 30 of the 170 endemic taxa growing in Turkey [2,3]. Some of the Orchidaceae species have been used as herbal medicines to treat headaches, dizziness, tetanus, and epilepsy. Some orchid species have been shown to exert sedative, anticonvulsive, and antiepileptic effects, as listed in the oldest Chinese materia medica [4,5]. In recent years, the neuroprotective functions of some Orchidaceae species are one of their traditional uses [6]. It is also used in perfumes, cosmetics, and aromatherapy. The orchids used in sahlep production are usually adapted to terrestrial and mild climate regions. Sahlep is generally used as a traditional beverage or food additive. Its use is very common as a hot drink in Turkey, the Middle East, and Balkan countries during the wintertime [7,8]. Sahlep consumed as a drink is prepared by adding milk to sahlep powder and sugar [9]. Sahlep is also used as a stabilizer to produce Kahramanmaraş-type ice cream in Turkey [10]. In the literature, the protein contents of orchids are mentioned to be in the range of 3.11%–4.95% [11]; thus, it is considered as a valuable food source. It has been reported that, depending on the species used, sahlep contains 17.7%–54.6% of glucomannan, 5.44%–38.7% of starch, and 0.5%–1% of nitrogenous substances based on dry material; the proportion of glucomannan was found to be higher than that of the starch of other orchid species.Glucomannans are carbohydrate polymers consisting of 1,4- *β* -D-mannose and 1,4- *β* -D-glucose units [12].Glucomannan is soluble fiber which delays gastric emptying time and provides a feeling of fullness, thus playing a role in appetite control. It is used as a natural laxative as it can increase stool volume without damaging the colonic microecology [13,14]. Studies show that it regulates intestinal microbiota and reduces the risk of colorectal cancer [14,15]. In addition, it ensures the removal of bile acids by feces. This situation can be evaluated as cholesterol excretion. Studies support the claim that glucomannan lowers blood cholesterol level [16]. Clinical trials of glucomannans used 3 g/day [17]. It is known that supplementary food products containing glucomannan can help control weight and fight the urge to overeat. Thus, *Serapias* orchids could be used as a healthy food supplement.

In the literature, there has been limited work related to the volatile constituents (EO, SPME, and n-hexane extract) of some *Serapias* species ( *Serapias vomeracea* (Burm. F.) B.; *Serapias vomeracea* subsp. *longipetala* (Ten.) H. Baumann & Künkele; *Serapias cordigera* L. subsp. *cordigera* ; *Serapias cordigera* subsp. *lucana* R. Lorenz & V.A. Romano; *Serapias parviflora* Parl.; *Serapias lingua* L.; *Serapias lingua* L.) [18–20]. The essential oil of *S. vomeracea* has been shown to have dose-dependent antioxidant effects through DPPH assay [18]. Some orchid species are known to exhibit antimicrobial and antioxidant biological activity [18,21]. However, to the best of our knowledge from the literature survey, no data about chemical compositions and biological activities of *S.orientalis* subsp. *orientalis* (Greuter) H.Baumann & Künkele have been reported to date. The subject of this study is to investigate the effect of different extraction methods. Therefore, in this paper, we report various volatile constituents of EO, SPME, and SPMEs of *n* -hexane, methanol, and water extracts analyzed with GC-FID/MS.Antimicrobial activities of the extracts ( *n* -hexane, methanol, and water) and EO against 9 microorganisms are evaluated. In addition, porcine pancreatic lipase (PPL) enzyme activity of EO and solvent extracts was investigated. The results of antimicrobial and porcine pancreatic lipase activities of EO and the solvent extracts ( *n* -hexane, methanol, and water) may be useful in foods, pharmaceuticals, and other industries.

## 2. Materials and methods

### 2.1. Plant materials

*S. orientalis* subsp. *orientalis* (245 g, wet) was harvested from the Karadeniz Technical University Campus (Ortahisar, Trabzon, Turkey) at an elevation of 130 m a.s.l. in May 2018. The plant was authenticated by Prof. Salih Terzioğlu [1–3]. A voucher specimen was deposited in the Herbarium of the Faculty of Forestry, KATO (KATO: 19322), Karadeniz Technical University, Trabzon, Turkey.

### 2.2. Chemicals and reagents

Analytical-grade solvents ( *n* -hexane, methanol, andacetonitrile), *p* -nitrophenyl butyrate ( *p* -NPB), Tris-HCl, and enzyme (crude porcine PL type II) were purchased from Sigma-Aldrich Corp. (St. Louis, MO, USA) and used without purification.

### 2.3. Hydrodistillation (HD) procedure for the isolation of EO

The fresh aerial part of *Soo* (115 g) was ground into small pieces and subjected to hydrodistillation (HD) using a modified Clevenger-type apparatus with a cooling bath (–15 °C) system (3h) [yield (w/w): 0.09%]. The obtained oil was extracted with *n* -hexane (0.5 mL), dried over anhydrous Na _2_ SO _4_ , and kept in sterilized dark glass bottles in the refrigerator at 4 °C prior to analysis.

### 2.4. Solvent extractions

The fresh plant (120 g) was ground into small pieces using a plant mill. Blended material was weighed (40 g each sample) into 3 different flasks (100 mL) and extracted 3 times with analytical-grade *n* -hexane, methanol, and water solvents (25 mL × 3; 12 h each). After the suction filtration, the same extracts were combined and evaporated at 40 °C to produce crude *n* -hexane (0.0445 g) and methanol (0.7356 g) extracts. The water extract was lyophilized to produce crude water extract (0.2470 g).

### 2.5. Gas chromatography-mass spectrometry (GC-FID/MS)

EO analysis was carried out using a Shimadzu QP2010 ultra GC-FID/MS, Shimadzu 2010 plus FID fitted with a PAL AOC-5000 plus auto sampler, and Shimadzu Class-5000 Chromatography Workstation software (Shimadzu Corp., Kyoto, Japan). The separation was analyzed by means of a Restek Rxi-5MS capillary column (30 mm × 0.25 mm × 0.25 μm) (Restek Corp., Bellefonte, PA, USA). The essential oil injections to GC-FID/MS wereperformed in split mode (1:30) at 230 °C. The essential oil solution (1 μL) in *n* -hexane (HPLC grade) was injected and analyzed with the column held initially at 60 °C for 2 min and then increased to 240 °C with a 3 °C/min heating ramp. The oven program was as follows: the initial temperature was 60 °C for 2 min, which was increased to 240 °C for3 min; the final temperature of 250 °C was held for 4 min. Helium (99.999%) was used as the carrier gas, with a constant flowrate of 1 mL/min. Detection was implemented in electronic impact mode (EI); ionization voltage was fixed at 70 eV and scan mode (40–450 *m/z* ) was used for mass acquisition. Each sample was analyzed and the mean of each reported.

### 2.6. Solid phase microextraction (SPME)

The blended fresh plant (2 g), *n* -hexane (0.0241 g), methanol (0.3802), and water extracts (0.1450) of *Soo* were placed into a sealed SPME vial (10 mL) with a silicone-rubber septum cap, then submitted to a solid-phase microextraction device (Supelco Inc., Bellefonte, PA, USA). A DVB/Carboxen/PDMS coating fiber was used to obtain volatile components. The SPME fibers were conditioned for 5 min at 250 °C in the GC injector. Extraction was achieved with magnetic stirring at 80 °C using an incubation time of 5 min and an extraction time of 10 min. Fibers with extracts of volatile compounds were subsequently injected into the GC injector. GC–FID/MS analysis was performed using a Shimadzu QP2010 Ultra mass selective detector attached to the 2010 Plus chromatograph. The carrier gas used was helium, at a flow rate of 1 mL/min. The injection was performed in split mode (1:30) at 230 °C. Samples were analyzed and the results recorded. The temperature, incubation, and extraction times were set according to the reported experiment [22].

### 2.7. Identification of volatile constituents

Retention indices of the volatile components of *Soo* were determined by the Kovats method using *n* -alkanes (C _6_ –C _32_ ) as standards [23–27].Volatile compounds were identified by comparison with literature RI [28–36]and MS, compared to existing analytical standards, and by matching mass spectral libraries (NIST, Wiley7NL, FFNSC1.2, and W9N11).

### 2.8. Antimicrobial activity assessment (agar-well diffusion method)

All test microorganisms were obtained from the Hıfzıssıhha Institute of Refik Saydam (Ankara, Turkey) and were as follows: *Escherichia coli* ATCC35218, *Yersinia pseudotuberculosis* ATCC911, *Pseudomonas aeruginosa* ATCC43288, *Enterococcus faecalis* ATCC29212, *Staphylococcus aureus* ATCC25923, *Bacillus cereus* 709 Roma, *Mycobacterium smegmatis* ATCC607, *Candida albicans* ATCC60193, and *Saccharomyces cerevisiae* RSKK 251. The antimicrobial screening test using the agar-well diffusion method as adapted was used earlier [37,38]. Each microorganism was suspended in brain heart infusion (BHI) broth (Difco Laboratories Inc., Detroit, MI, USA) and diluted to approximately 106 colony forming units (cfu) per mL. They were flood-inoculated onto the surface of BHI agar and Sabouraud dextrose agar (SDA) (Difco Laboratories Inc.), and then dried. For *C. albicans* , SDA was used. Five-millimeter–diameter wells were cut from the agar using a sterile cork-borer, and 50μL of the extract substances was delivered into the wells. The plates were incubated for 18 h at 35 °C. Antimicrobial activity was evaluated by measuring the zone of inhibition against the test organism. Extract stock solutions were prepared at different concentrations (10,000–155,400 μg/mL) according to the amount of material obtained. A 1/10 dilution of each solvent was used as a control.

### 2.9. Minimal inhibition concentration (MIC) assay

The antimicrobial properties of EO, *n* -hexane, methanol, and water extracts of *Soo* were investigated quantitatively in respective broth media by using double microdilution; the minimal inhibition concentration (MIC) values (μg/mL) were examined [37,38] and used in our previous work [25,26]. The antibacterial and antifungal assays were carried out in Mueller–Hinton broth (MH) (Difco Laboratories Inc.) at pH. 7.3 and buffered yeast nitrogen base (Difco Laboratories Inc.) at pH 7.0, respectively. The microdilution test plates were incubated for 18 h at 35 °C. Brain heart infusion broth (BHI) (Difco Laboratories Inc.) was used for *M. smegmatis* , and incubated for 48–72 h at 35 °C. The MIC was defined as the lowest concentration that showed no growth. Ampicillin (10 mg/mL), streptomycin (10 mg/mL), and fluconazole (2 mg/mL) were used as standard antibacterial and antifungal drugs, respectively. The 1/10 dilution of each solvent was used as a control.

### 2.10. Lipase inhibitory effect assay

EO, *n* -hexane, methanol, and water extracts of the *Soo* species studied were evaluated for lipase (PPL) inhibitory assay by the modified method, using *p* -nitrophenyl butyrate ( *p* -NPB) as the substrate [39–41]. First, all fractions were dissolved with buffer solution (0.1 M Tris-HCl buffer, pH = 8.0) in 25, 50, 100, and 200 µg/mL concentrations. Orlistat, used as a positive control, was prepared in 6.25, 12.5, 25, 50, and 100 µg/mL concentrations. The test procedure was designed as I, II, III, and IV wells, described as follows: I, 90 enzyme solution (crude porcine PL type II, 200 units/mL), 5 µL substrate solution (10 mM *p* -NPB in acetonitrile), 5 µL buffer solution (0.1 M Tris-HCl buffer, pH = 8.0); II, 90 µL enzyme solution, 10 µL buffer solution; III, 90 µL enzyme, 5 µL sample solution, 5 µL substrate solution; IV, 90 µL enzyme solution, 5 µL sample solution, 5 µL buffer solution. The plates were incubated at 37 °C for 15 min. Following incubation, substrate solution (10 mM *p* -NPB in acetonitrile) was added to each well. The plates were incubated at 37°C for 15 min, and the absorbance of the reaction mixture was determined spectrophotometrically at 405 nm in a 96-well microplate using a spectrophotometer (SPECTROstar Nano, BMG LABTECH Inc., Cary, NC, USA). Each sample was tested in triplicate. The percentage of PPL inhibitory effect was calculated using the following equation:

PPL inhibition (%) = [[(I– II)– (III–IV)]/(I–II)]×100.

Afterwards, the half maximal inhibitory concentration (IC _50_ ) values for PPL were calculated graphically.

## 3. Results and discussion

### 3.1. Analysis of bioactive compounds

The VOCs in EO, SPME, and SPME of extracts ( *n* -hexane, methanol, and water) of fresh *Soo* were investigated by GC-FID/MS analysis. The chemical names of the identified volatile constituents of *Soo* , together with their retention indices and percentages, are given in Table 1, where the compounds have been listed in the order of elution on the Restek Rxi-5MS column used [42]. Volatile compounds were identified by comparison of the registered mass spectrum libraries (NIST, Wiley7NL, FFNSC1.2, and W9N11), and by using the Kovats index [23–36].

**Table 1:** Identified VOCs from the aerial parts of fresh S. orientalis subsp. orientalis growing in Turkey.

Compounds	RIa	RIb	Lit.	Percentage peak areac				
				EO	SPME	A1	A2	A3
Mehylbenzene	787	792	35	1.48	-	-	-	-
Capronaldehyde	801	804	28	37.01	2.1	3.0	10.3	-
2(E)-hexenal	861	860	33	23.19	-	-	-	-
Heptanal	906	906	28	2.21	10.4	7.9	-	0.6
2(E)-heptenal	959	957	32	-	-	-	2.1	-
Myrcene	998	995	28	-	0.1	0.4	-	1.1
Octanal	998	999	28	-	-	3.6	-	-
Decane	1000	1001	28	9.03	-	-	-	-
Limonene	1031	1030	28	-	76.5	41.7	61.3	45.3
Heptanoic acid	1078	1076	36	-	-	0.3	-	-
Pelargonaldehyde	1100	1102	28	19.05	7.0	21.5	12.3	-
Caprylic acid	1167	1164	28	-	-	0.1	-	-
Decanal	1201	1201	28	-	-	5.2	7.5	-
Methyl nonanoate	1227	1219	31	-	0.6	-	-	-
α-methoxy-p-cresol	1306	1298	34	-	-	-	-	52.9
Undecanal	1305	1303	28	2.85	3.2	15.1	4.2	-
Dodecanal	1408	1403	28	-	-	0.7	-	-
Tridecanal	1509	1503	28	-	-	0.4	2.0	-
Hexadecanal	1817	1811	29	3.88	-	-	-	-
Palmitic acid	1959	1954	28	0.50	-	-	-	-
Octadecanal	2021	2014	30	0.40	-	-	-	-
Ethyl oleate	2168	2165	31	0.37	-	-	-	-
Chemical class				Total % and number of compounds				
Monoterpene				-	76.6:2	42.1:2	61.3:1	46.4:2
Aldehyde				88.6:7	22.7:4	57.4:8	38.4:6	0.6:1
Aliphatic				9.0:1	-	-	-	-
Aromatic				1.4:1	-	-	-	52.9:1
Acid				0.5:1	-	0.4:2	-	-
Ester				0.4:1	0.6:1	-	-	
Total				99.9:11	99.9:7	99.9:12	99.7:7	99.9:4

^a^ Literature RI; bRI calculated from retention times relative to that of n-alkane (C _6_ -C _32_ ) series; cPercentage peak area obtained by FID peak-area normalization; EO: Essential oil; Hydrodistillation by Clevenger method of S. orientalis subsp. orientalis ; SPME: SPME of S. orientalis subsp. orientalis; A1: SPME of hexane extract; A2: SPME of methanol extract; A3: SPME of water extract.

GC-FID/MS analysis for the EO of *Soo* gave 11 natural compounds totaling 99.97%. EO of *Soo* was characterized by a high percentage of aldehydes (88.6%), with capronaldehyde (37.01%), 2( *E* )-hexenal (23.19%) pelargonaldehyde (19.05%), and decane (9.03%) as the major constituents. The terpene-type compound was not detected in the essential oil ofthe *Soo.* The composition of EO of *Soo* differed from that of EO of the other *Serapias* species. The main represented volatile compounds were reported to be saturated hydrocarbons (53.29%) in EO of *S. vomeracea* and pentacosane (17.59%); *n* -tricosane (14.21%), *n* -heneicosane (5.68%), and *n* -heptacosane (4.99%) were the most abundant compounds from the Italian orchid [18].

SPME GC-FID/MS analysis for the fresh plant and solvent extract ( *n* -hexane, methanol, and water) of *Soo* revealed 7, 12, 7, and 4 natural compounds totaling 99.7%–99.9%. Limonene (76.6%, 41.8%, and 61.6%) was found to be the major compound in the SPME of the fresh plant and the SPMEs of the *n* -hexane and methanol extracts of *Soo* , respectively. SPME of the water extract revealed *α* -methoxy- *p* -cresol (52.9%) as the main component. Limonene is widely used as a flavor and fragrance additive in perfumes, beverages, and cleaning products [43]. Considering its medicinal potential and cosmetic uses, it is important that *Soo* might be a good source for limonene. Monoterpenes (76.6% and 61.3%) werethe major constituent in the SPME of the fresh plant and the SPME of methanol extract. Aldehydes and aromatic compounds accounting for 57.4% and 52.9% of the SPMEs of *n* -hexane and water extracts respectively were the most abundant constituents (Table 1). Major constituents were found to be limonene (76.5%, 41.7%, 61.3%), pelargonaldehyde (7.0%, 21.5%, and 12.3%), undecanal (3.2%, 15.1%, and 4.2%), and heptanal (10.4% and 7.9%) in the SPMEs of *n* -hexane and methanol extracts, respectively. *α* -methoxy- *p* -cresol (52.9%) and limonene (45.3%) were the most abundant constituents of the SPME of the water extract.

In the literature, the relevant components of *Serapias* species were listed as α-amorphene (28.85%), ethyl oleate (15.33%), heneicosane (4.14%), heptadecane (3.96%), 2-heptadecanone (3.42%), and ethyl elaidate (3.39%) in *S. cordigera* [19]; α-amorphene (38.06%), pentadecane (11.13%), propyl undecanoate (5.15%), heptadecane (4.55%), ethyl elaidate and oleate (3.47% and 2.66%, respectively), ethyl stearate (3.33%), and 8-heptadecene (3.02%) in *S. cordigera* subsp. *Lucana* [19]; pentadecane (25.43%), 5-nonadecene (14.48%), 1-nonadecene (5.16%), 3-heptadecene (4.33%), benzyl benzoate (4.18%), 2-undecanone (3.31%), and octadecane (3.24%) in *S. vomeracea* [19]; heptadecane (11.70%), 3-heptadecene (8.73%), isopropyl myristate (7.80%), pentadecane (7.41%), nonadecane (6.38%), isopropyl palmitate (4.86%), octadecane (4.49%), and benzyl benzoate (3.80%) in *S. lingua* [19]; pentadecene (7.05%), hexadecane (3.96%), heptadecane (6.38%), nonadecane (4.28%), and ethyl elaidate (4.31%) in *S. parviflora* [19]. In that reported work, most of the volatile constituents were mentioned to be alkanes and alkene-type compounds. In another work, GC-MS analysis of *n* -hexane extract of *Serapias* species ( *S. lingua* , *S. parviflora* , *S. cordigera* , and *S. vomeracea* ) revealed C20–C29 alkanes and alkenes [20]. In our study, *n* -decane was the only hydrocarbon found in the EO of *S. orientalis* subsp. *orientalis.* However, aldehydes (88.6%) in the EO and limonene (76.5%, 41.7%, 61.3%, and 45.3%) and *α* -methoxy- *p* -cresol (52.9%) respectively in the SPMEand SPMEs of *n* -hexane, methanol, and water extracts of fresh *Soo* were the major constituents differing from other *Serapias* species in previous work. Comparison of the present results with the literature suggests that limonene and *α* -methoxy- *p* -cresol might be used as taxonomical markers for the future classification of *S. orientalis* . The variations in the VOCs in *Serapias* species may be due to environmental, storage, and analysis conditions. The dominant compounds of the SPME and SPMEs of extracts of *Soo* showed similarities, since all of them are from the same plant. However, they also had several uncommon compounds due to the differences in the extracts.

### 3.2. Antimicrobial and lipase enzyme activities

Antimicrobial activity of EO and solvent extracts ( *n* -hexane, methanol, and water) obtained from *Soo* were screened by using the agar well diffusion method with the microorganisms seen in Table 2. In general, EO and *n* -hexane extract showed moderate antimicrobial activities with inhibition zones of 8/12 mm, 9/12 mm, and 20/8 mm against *S. aureus* , *B. cereus* , and *M. smegmatis* , respectively. Water extract gave better activity against *E. faecalis* , *S. aureus* , and *B. cereus* with 15-, 20-, and 10-mm inhibition zones, respectively. Methanol extract showed moderate antimicrobial activity against all tested microorganisms except for *C. albicans* and *S. cerevisiae.* The results indicate that antimicrobial activity of EO, *n* -hexane, and water extracts are more susceptible to gram-positive bacteria. The MIC of the methanol extract of *S. orientalis* subsp. *orientalis* was within the range of 121.4–3885.0 µg/mL against *E. coli* , *Y. pseudotuberculosis* , *P.aeruginosa* , *E. faecalis* , *S. aureus* , *B. cereus* , and *M. smegmatis* . EO and solvent extracts which contain limonene, *α* -methoxy- *p* -cresol, and aldehydes as major components of the samples have been reported to have antibacterial properties [43–46]. Therefore, the bactericidal activity of the EO and solvent extracts obtained from *Soo* may be mainly related to these compounds. Other compounds which were also present in the samples, including capronaldehyde, 2( *E* )-heptenal, octanal, heptanoic acid, pelargonaldehyde, caprylic acid, decanal, methyl nonanoate, undecanal, dodecanal, tridecanal, hexadecanal, palmitic acid, octadecanal, and ethyl oleate, have been reported to have antibacterial properties, and may also collectively have a remarkable contribution to the bactericidal activities of the EO and solvent extracts. Recently, a series of aliphatic aldehydes from olive has been shown to exhibit noticeable activity against several microfungal and bacterial strains [45,46].The antibacterial activity investigation showed variations which may be due to factors such as composition and concentration of the EO and solvent extracts. However, the investigated methanol extract of this plant with its major compound of *α* -methoxy- *p* -cresol (52.9%) and limonene (45.9%) showed the highest antibacterial activity against *S. aureus* , with 121.4 µg/mL MIC value [38].The greatest lipase enzyme inhibitions [39–41] were obtained for EO and *n* -hexane extract of *Soo* with IC _50_ values of 59.87 µg/mL and 64.03 µg/mL, respectively (Figure).

**Table 2:** Screening for the antimicrobial activity of EO and the solvent extracts of S. orientalis subsp. orientalis

Extracts	Stock sol. (µg/mL)		Microorganisms, inhibition zone (mm) and minimal inhibition concentration (MIC, μg/mL)								
			Ec	Yp	Pa	Ef	Sa	Bc	Ms	Ca	Sc
EO	23000	mm	-	-	-	-	8	9	20	-	-
		MIC	-	-	-	-	1150	1150	79.5	-	-
Hexane	10000	mm	-	-	-	-	12	12	8	-	-
		MIC	-	-	-	-	125	125	250	-	-
Methanol	155400	mm	11	8	19	6	21	6	8	-	-
		MIC	971.3	1942	489.6	3885	121.4	3885	3885	-	-
Water	52000	mm	-	-	-	15	20	10	-	-	-
		MIC	-	-	-	162.5	81.25	650	-	-	-
Amp.	10	mm	10	10	18	10	35	15	-	-	-
		MIC	10	18	128	35	10	15			
Strep.	10	mm							35		
		MIC							4		
Flu	5	mm								25	25
		MIC								8	8

Ec: Escherichia coli, Yp: Yersinia pseudotuberculosis, Pa: Pseudomonas aeruginosa, Sa: Staphylococcus aureus, Ef: Enterococcus faecalis, Bc: Bacillus cereus 702 Roma, Ms: Mycobacterium smegmatis, Ca: Candida albicans, Sc: Saccharomyces cerevisiae, Amp.: Ampicillin, Strep.: Streptomycin, Flu.: Fluconazole, (-): no activity.

**Figure:**
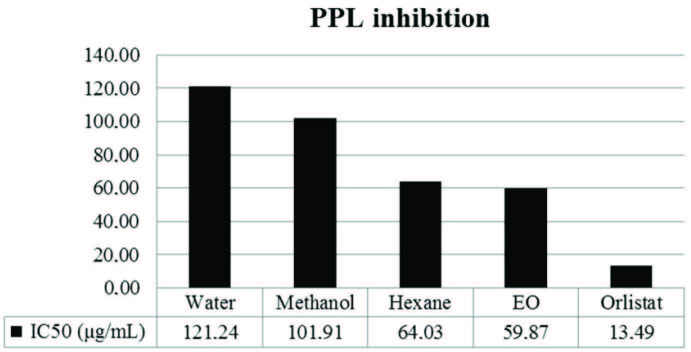
Porcine pancreatic lipase inhibitory effect assay of EO and the solvent extracts of S. orientalis subsp. orientalis.

## 4. Conclusion

VOC composition of *Soo* has been analyzed, and their antimicrobial and lipase enzyme activities investigated for the first time. Limonene was the major compound of allextracts (SPME, SPMEs of EO, *n* -hexane, and methanol extracts) obtained from fresh *Soo* within the range of 41.7% to 76.5%, respectively. However, *α* -methoxy- *p* -cresol (52.9%) was found to be a major component only in the SPME of the methanol extract of the plant. This clearly showed that various extraction methods used in this work resulted in the identification of different components as in the literature. *n* -Hexanal was the major compound obtained in EO of fresh *Soo* . Aldehydes were found to be the major class of components (88.6%) in the EO. The amount of limonene and *α* -methoxy- *p* -cresol is so high that *Soo* could be a source for the production of these compounds. In general, the greatest activity of EO was against *M. smegmatis* with 79.5 µg/mL MIC value. According to the agar diffusion method, the most antimicrobial activity was observed against Gram-positive and Gram-negative bacteria in methanol extract, which had better antimicrobial activity against *P. Aeruginosa* and *S. aureus* with 19-mm and 21-mm inhibition zones, respectively. The MIC values were found to be 489.6 µg/mL and 121.4 µg/mL, respectively. The EO of the *Soo* was found to be most effective against the lipase enzyme (59.87 µg/mL, IC _50_ ). Therefore, the overall results of antimicrobial and lipase enzyme activities suggest that EO and solvent extracts of *Soo* may be promising prospects for pharmaceutical, food, and other industrial applications. In further study, activity guided isolation and purification could be carried out on *Soo.*
